# Supplementary material for: Nuclear Factor Erythroid 2 Regulates Human HSC Self-Renewal and T Cell Differentiation by Preventing NOTCH1 Activation
Source: Stem Cell Reports. 2017 Jun 22;9(1):5–11. doi: 10.1016/j.stemcr.2017.05.027 (PMC5511106; doi:10.1016/j.stemcr.2017.05.027)
Supplement: Document S1. Figures S1 and S2 [file mmc1.pdf]

Stem Cell Reports, Volume *9*

Supplemental Information

Nuclear Factor Erythroid 2 Regulates Human HSC Self-Renewal and T Cell Differentiation by Preventing NOTCH1 Activation

Alessandro Di Tullio, Diana Passaro, Kevin Rouault-Pierre, Sukhveer Purewal, and Dominique Bonnet

Figure S1

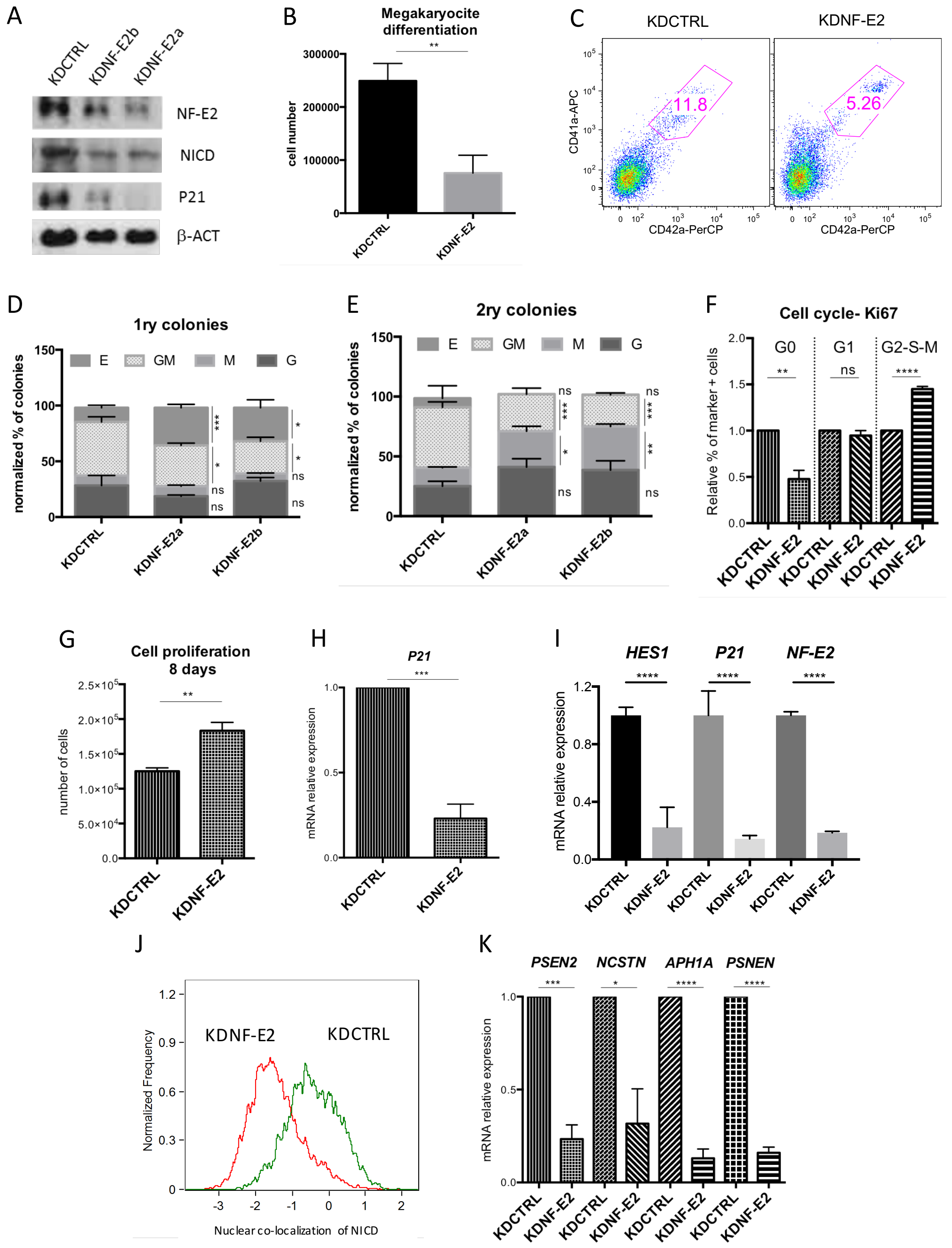
A
KDCTRL
KDNF-E2b
KDNF-E2a
NF-E2
NICD
P21
β-ACT
B
Megakaryocite differentiation
**
cell number
300000
200000
100000
0
KDCTRL
KDNF-E2
C
KDCTRL
KDNF-E2
CD41a-APC
11.8
5.26
CD42a-PerCP
CD42a-PerCP
D
1ry colonies
normalized % of colonies
E
GM
M
G
KDCTRL
KDNF-E2a
KDNF-E2b
E
2ry colonies
normalized % of colonies
F
Cell cycle- Ki67
Relative % of marker + cells
G0
G1
G2-S-M
G
Cell proliferation
8 days
number of cells
H
P21
mRNA relative expression
I
HES1
P21
NF-E2
mRNA relative expression
J
KDNF-E2
KDCTRL
Normalized Frequency
Nuclear co-localization of NICD
K
PSEN2
NCSTN
APH1A
PSNEN
mRNA relative expression

Figure S1. Knock-down of *NF-E2* in HSPCs reduces megakaryocyte differentiation and favors cell proliferation over self-renewal by inhibition of NOTCH1 activation, Related to Figure 1.

(A) Western Blot showing the expression of P21, Notch 1 NCID and NF-E2 in HSPCs 6 days after transduction. β*-ACT* has been used as a control.

(B) Quantification of $CD41a^{+}CD42a^{+}$ cells in transduced HSPCs 2 weeks after the beginning of megakaryocyte differentiation. **(C)** FACS analysis showing the percentage of CD41a and CD42a as in A. **(D)** CFC assay using HSPCs transduced with KDCRTL or two independent KDNF-E2 (N=3. **(E)** Secondary CFC assay using cells taken from CFU in C (N=3). **(F)** Cell cycle analysis by FACS using Ki67 and DAPI to identify proportion of cells in G0, G1 and S/G2/M phases of HSPCs 8 days after transduction (N=3). **(G)** Quantification of cell proliferation by cell counting of HSPCs 8 days after transduction (N=3). **(H)** RNA expression of *P21* in HSPCs 4 days after transduction. β*-ACT* was used as a control gene (N=3). **(I)** RNA expression of *HES1*, *P21*, and *NF-E2* in MOLT4 transduced with KDCTRL and KDNF-E2. β*-ACT* was used as a control gene (N=3). **(J)** ImageStream^{x} analysis showing the frequency of NICD nuclear co-localization in KDNF-E2 (red) and KDCTRL (green) transduced MOLT4 cells as shown in Figure 1J. **(K)** RNA expression of *PSEN2*, *NCSTN*, *APH1A* and *PSNEN* in HSPCs 4 days after transduction. β-*ACT* was used as a control gene (N=3). ns= not significant, * $p<0.05$; ** $p<0.001$ and *** $p<0.0001$.

Figure S2

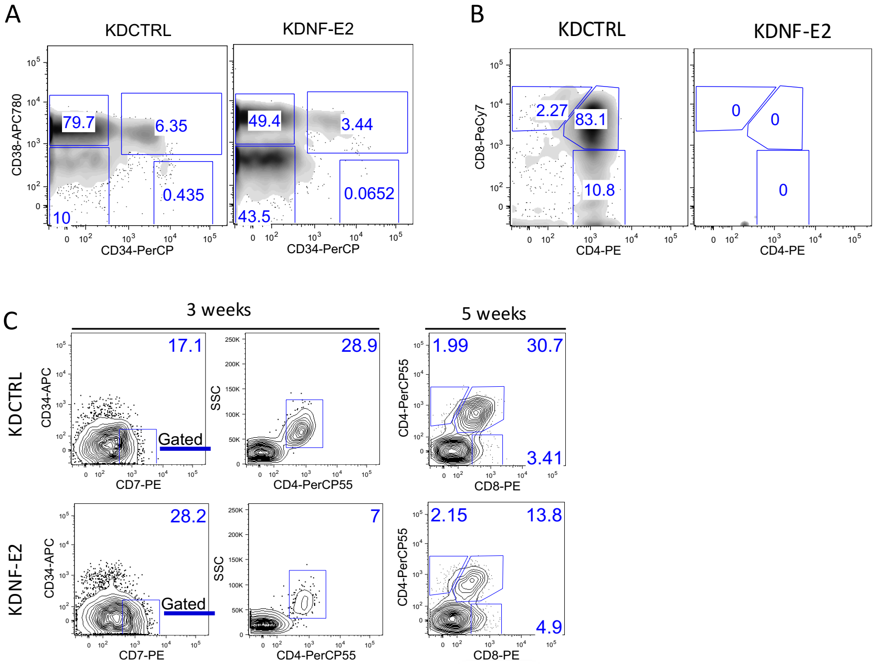

Figure S2. Knock-down of *NF-E2* in HSPCs impairs T cell development, Related to Figure 2.

(A) FACS analysis showing the percentage of human CD38/CD34 cells in the bone marrow of mice transplanted with transduced HSPCs at 18 weeks after injection (N=5). **(B)** Representative FACS analysis showing the percentage of human CD4 and CD8 cells in thymus of mice transplanted with transduced HSPCs at 18 weeks after injection. **(C)** Representative FACS analysis showing the percentage of pre-T cell makers 3 weeks (left panel) and mature T cell markers 5 weeks (right panel) after the induction of T cell differentiation.
