# Supplementary material for: Nuclear Factor Erythroid 2 Regulates Human HSC Self-Renewal and T Cell Differentiation by Preventing NOTCH1 Activation
Source: Stem Cell Reports. 2017 Jun 22;9(1):5–11. doi: 10.1016/j.stemcr.2017.05.027 (PMC5511106; doi:10.1016/j.stemcr.2017.05.027)
Supplement: Document S2. Article plus Supplemental Information [file mmc2.pdf]

Stem Cell Reports
Report
OPEN ACCESS

# Nuclear Factor Erythroid 2 Regulates Human HSC Self-Renewal and T Cell Differentiation by Preventing NOTCH1 Activation

Alessandro Di Tullio,[1] Diana Passaro,[1] Kevin Rouault-Pierre,[1] Sukhveer Purewal,[2] and Dominique Bonnet[1,*]
[1]Haematopoietic Stem Cell Laboratory
[2]Flow Cytometry Laboratory
The Francis Crick Institute, 1 Midland Road, London NW1 1AT, UK
*Correspondence: dominique.bonnet@crick.ac.uk
http://dx.doi.org/10.1016/j.stemcr.2017.05.027

## SUMMARY

Nuclear factor erythroid-derived 2 (NF-E2) has been associated with megakaryocyte maturation and platelet production. Recently, an increased in NF-E2 activity has been implicated in myeloproliferative neoplasms. Here, we investigate the role of NF-E2 in normal human hematopoiesis. Knockdown of NF-E2 in the hematopoietic stem and progenitor cells (HSPCs) not only reduced the formation of megakaryocytes but also drastically impaired hematopoietic stem cell activity, decreasing human engraftment in immunodeficient (NSG) mice. This phenotype is likely to be related to both increased cell proliferation (p21-mediated) and reduced Notch1 protein expression, which favors HSPC differentiation over self-renewal. Strikingly, although NF-E2 silencing in HSPCs did not affect their myeloid and B cell differentiation *in vivo*, it almost abrogated T cell production in primary hosts, as confirmed by *in vitro* studies. This effect is at least partly due to Notch1 downregulation in NF-E2-silenced HSPCs. Together these data reveal that NF-E2 is an important driver of human hematopoietic stem cell maintenance and T lineage differentiation.

## INTRODUCTION

Nuclear factor erythroid-derived 2 (NF-E2) is a well-known transcription factor important for megakaryocyte maturation and platelet production (Shivdasani et al., 1995). NF-E2 knockout mice exhibit absence of circulating platelets, leading to hemorrhage and death in most animals, and megakaryocyte differentiation arrest followed by profound thrombocytopenia (Lecine et al., 1998). Notably, NF-E2 is highly expressed in murine erythroid and megakaryocytic lineages, but interestingly also in hematopoietic stem cells (HSCs) (Andrews et al., 1993; Di Tullio et al., 2011). Due to the high lethality of NF-E2 knockout mice, its role in the hematopoietic stem compartment in mice has never been described.

In a human clinical setting, overexpression of NF-E2 was first identified in polycythemia vera (PV) patients, and later extended to all myeloproliferative neoplasms (Goerttler et al., 2005; Wang et al., 2010), disorders characterized by overproduction of erythroid cells and often megakaryocytes and platelets. Overexpression of human NF-E2 in the murine hematopoietic system have been shown to cause an expansion of early HSCs and progenitor cells and the development of a myeloproliferative neoplasm (Kaufmann et al., 2012). Moreover, it has recently been reported that nuclear factor erythroid 2-related factor 2 (Nrf2), known to have DNA-binding specificities similar to those of NF-E2 (Motohashi et al., 2010), plays a regulatory role in several aspects of HSC homeostasis (Tsai et al., 2013). These observations suggest that NF-E2 might also have a role in HSCs.

## RESULTS AND DISCUSSION

To provide a comprehensive landscape in human hematopoiesis, we studied NF-E2 gene expression throughout the hematopoietic system using HemaExplorer (Bagger et al., 2013) and confirmed by RT-PCR that it was also expressed in the stem cell compartment (Figure 1A). Thereafter, we knocked NF-E2 down in hematopoietic stem and progenitor cells (HSPCs: $Lin^{-}/CD34^{+}/CD38^{-}$) derived from human umbilical cord blood (hUCB) using two independent NF-E2 knockdown plasmids (KDNF-E2a and KDNF-E2b) and one knockdown control (KDCTRL). We checked NF-E2 expression 4 days after HSPC transduction and obtained a significant decrease at the mRNA level (Figure 1B), which was confirmed at the protein level 6 days after transduction (Figures 1C and S1A). As expected, lack of NF-E2 in HSPCs led to a reduction in megakaryocyte differentiation *in vitro*, as shown by cell counting (Figure S1B) and fluorescence-activated cell sorting (FACS) analysis (Figure S1C). To test the functionality of the *NF-E2*-silenced HSPCs, we performed a colony-forming cell (CFC) assay and observed a significant reduction in colony-forming units (CFU; Figure 1D). There was a significant increase in the relative proportion of erythroid colony-forming units (CFU-E), while the percentage of myeloid colony-forming units (granulocytes = CFU-G, macrophages = CFU-M, granulocyte-macrophages = CFU-GM) was unaffected (Figure S1D). This increase appeared paradoxical, as we expected NF-E2 downregulation to reduce erythroid differentiation, yet our data are in line with what has been previously described in an overexpression experiment, where NF-E2


© 2017 The Author(s).
This is an open access article under the CC BY license (http://creativecommons.org/licenses/by/4.0/).

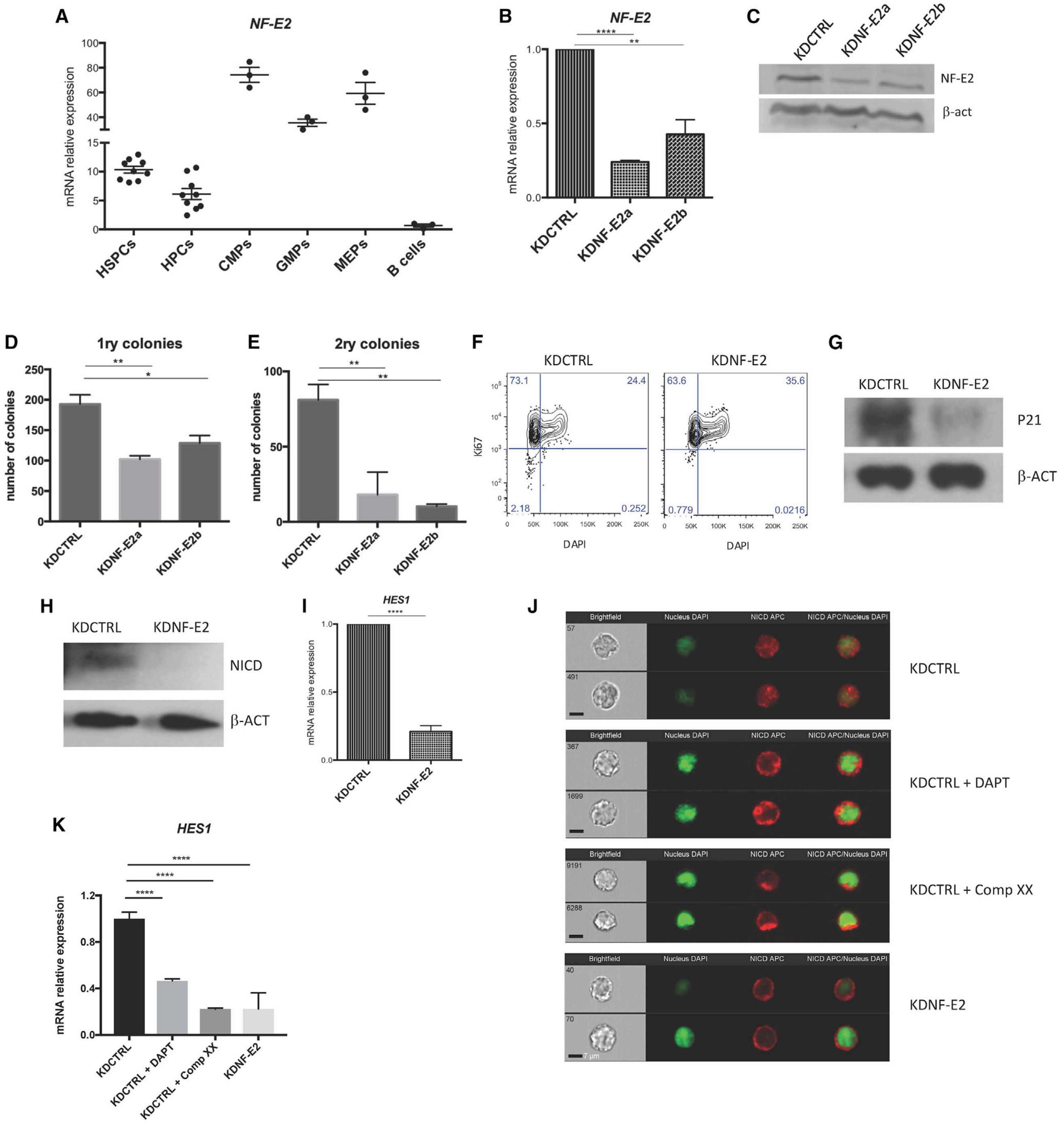


**Figure 1. Silencing *NF-E2* in HSPCs Affects Human HSC Self-Renewal *In Vitro***

(A) RNA expression of *NF-E2* in different human hematopoietic compartments. *β-ACT* was used as a control gene. HSPCs, hematopoietic stem and progenitor cells (n = 9); HPCs, hematopoietic progenitor cells (n = 9); CMPs, common myeloid progenitor cells (n = 3); GMPs, granulocyte monocyte progenitors (n = 3); MEPs, megakaryocyte-erythroid progenitor cells (n = 3); B cells (n = 3).

(B) RNA expression of *NF-E2* in HSPCs 4 days after transduction. *β-ACT* was used as a control gene (n = 3).

(C) Western blot showing the expression of NF-E2 in HSPCs 6 days after transduction. *β-ACT* was used as a control.

(D) CFC assay using HSPCs transduced with KDCRTL or two different KDNF-E2 (n =3).

(E) Secondary CFC assay using cells taken from CFU in (A) (n =3).

*(legend continued on next page)*

overexpression in human CD34+ cells reduced the number of CFU-E, arguing that it delays erythroid maturation and retains erythroid progenitors in an immature stage with increased proliferation capacity (Mutschler et al., 2009). Next, we examined whether KDNF-E2 HSPCs displayed altered self-renewal capacity by replating colonies (Figure 1E). The reduction of total CFU was even stronger in secondary colonies and was associated with a further decrease in CFU-GM and an increase in CFU-M (Figure S1E). In light of the similar effects generated by the two *NF-E2* knockdown plasmids, we continued the study using only KDNF-E2a (which we then simply identified as KDNF-E2). We hypothesized that decreased self-renewal capacity of HSPCs, highlighted by their reduced capacity to generate colonies, could be counterbalanced by an increase in cell proliferation. To investigate cell proliferation dynamics, we checked the cell-cycle status of HSPCs 8 days after KDNF-E2 by Ki67/DAPI staining, and noticed a decrease in $G_0$ and an increase in $G_2/M/_S$ phase *in vitro* (Figures 1F and S1F). We also observed a significant increase in cell number 8 days after NF-E2 silencing (Figure S1G) and a concurrent decrease in P21, a negative regulator of the $G_1/S$ cell-cycle transition at both the RNA level (Figure S1H) and the protein level (Figure 1I, day 8 and Figure S1A, day 6). It has been reported that NOTCH1 activation favors self-renewal over differentiation in murine HSCs (Stier et al., 2002). We therefore studied whether NF-E2 could interfere with Notch1. Interestingly, we observed a strong reduction of activated NOTCH1 (NOTCH intracellular domain [NICD]) in HSPCs 6 days after NF-E2 silencing (Figures 1H and S1A), and also detected downregulation of its downstream target *HES1* (Figure 1I). To further support this, we transduced human T-acute lymphoblastic leukemia (T-ALL) MOLT4 cells with KDNF-E2 and KDCTRL and induced NOTCH1 activation by growing them on the δ1 receptor-expressing MS5 stroma layer (MS5-DL1). We compared the effect of KDNF-E2 with two known γ-secretase inhibitors ((S)-tert-butyl 2-((S)-2-(2-(3,5-difluorophenyl)acetamido)propanamido)-2-phenylacetate [DAPT] and compound XX). We confirmed in MOLT-4 that knockdown of NF-E2 significantly affects P21 and HES1 level (Figure S1I). We also observed a reduction of NICD nuclear localization by ImageStreamX analysis in MOLT4 cells when NF-E2 was silenced (Figures 1J and S1J) comparable with DAPT- and compound XX-treated cells (Figure 1J). We confirmed these results by a comparable reduction in the expression of *HES1* between KDNF-E2 and the two γ-secretase inhibitors (Figure 1K).

We discarded the hypothesis that NOTCH1 could be a direct target of NF-E2 because it was still detectable in the cytoplasm of KDNF-E2 MOLT4 cells, and the expression of full-length NOTCH1 was not affected by NF-E2 knockdown (data not shown). Since the γ-secretase complex activates NOTCH1 by proteolytic processing (Artavanis-Tsakonas et al., 1999), we hypothesized that NF-E2 might interfere with γ-secretase expression. Interestingly, we found downregulation of γ-secretase components when silencing NF-E2 in HSPCs (Figure S1K). These findings support our hypothesis that NF-E2 acts as a regulator of HSPC self-renewal and cell proliferation via at least its activation of NOTCH1.

To further explore this phenomenon *in vivo*, we exploited our NOD-SCID IL2R$\gamma^{null}$ (NSG) xenograft mouse model, injecting hUCB-derived HSPCs transduced with KDNF-E2 and KDCTRL, as depicted in Figure 2A. We observed a decrease in the level of human hematopoietic engraftment (human CD45 expression) in the KDNF-E2 mice cohort 18 weeks after HSPCs injection in three different hematopoietic tissues (bone marrow [BM], blood, and spleen; Figure 2B). More specifically, we noted an exhaustion of HSPCs (Lin$^-$/CD34$^+$/CD38$^-$) and HPCs (Lin$^-$/CD34$^+$/CD38$^+$), and a strong increase in differentiated cells, in the BM of mice injected with KDNF-E2 HPSCs (Figure S2A). To investigate whether the altered engraftment was caused by reduced HSPC self-renewal capacity, and not by defects in homing, we assessed the retention of HSPCs in the BM 4 days after injection and observed no homing impairment by intra-vital imaging of the calvarium BM (data not shown). Moreover, we could confirm the altered self-renewal capacity by performing CFC assay on KDNF-E2 and KDCTRL HSPC cells extracted

(F) Cell-cycle analysis by FACS using Ki67 and DAPI to identify proportion of cells in $G_0$, $G_1$, and $S/G_2/M$ phases in HSPCs 8 days after transduction. Western blot showing the expression of P21 (G) and Notch1 NCID (H) in HSPCs 6 days after transduction. β-Actin was used as a control.
(I) RNA expression of *HES1* in HSPCs 4 days after transduction (n =3).
(J) Representative ImageStreamX analysis showing localization of NICD (red) with the nucleus DAPI in green of MOLT4 transduced with KDCTRL or KDNF-E2 after being cultured on MS5-DL1 feeder layer. We used two γ-secretase inhibitors (DAPT and compound XX) as control. Two examples per condition (n =2). Scale bar is incorporated in the figure.
(K) RNA expression of *HES1* in MOLT4 transduced with KDCTRL and treated with two different γ-secretase inhibitors (DAPT and compound XX). MOLT4 transduced with KDNF-E2 were used as a control. *β-ACT* was used as a control gene (n =3).
Error bars indicate the SEM of data from replicate experiments. The significance of the difference between samples was confirmed using unpaired t tests with equal SD. *p < 0.05, **p < 0.001, ****p < 0.00001. See also Figure S1.

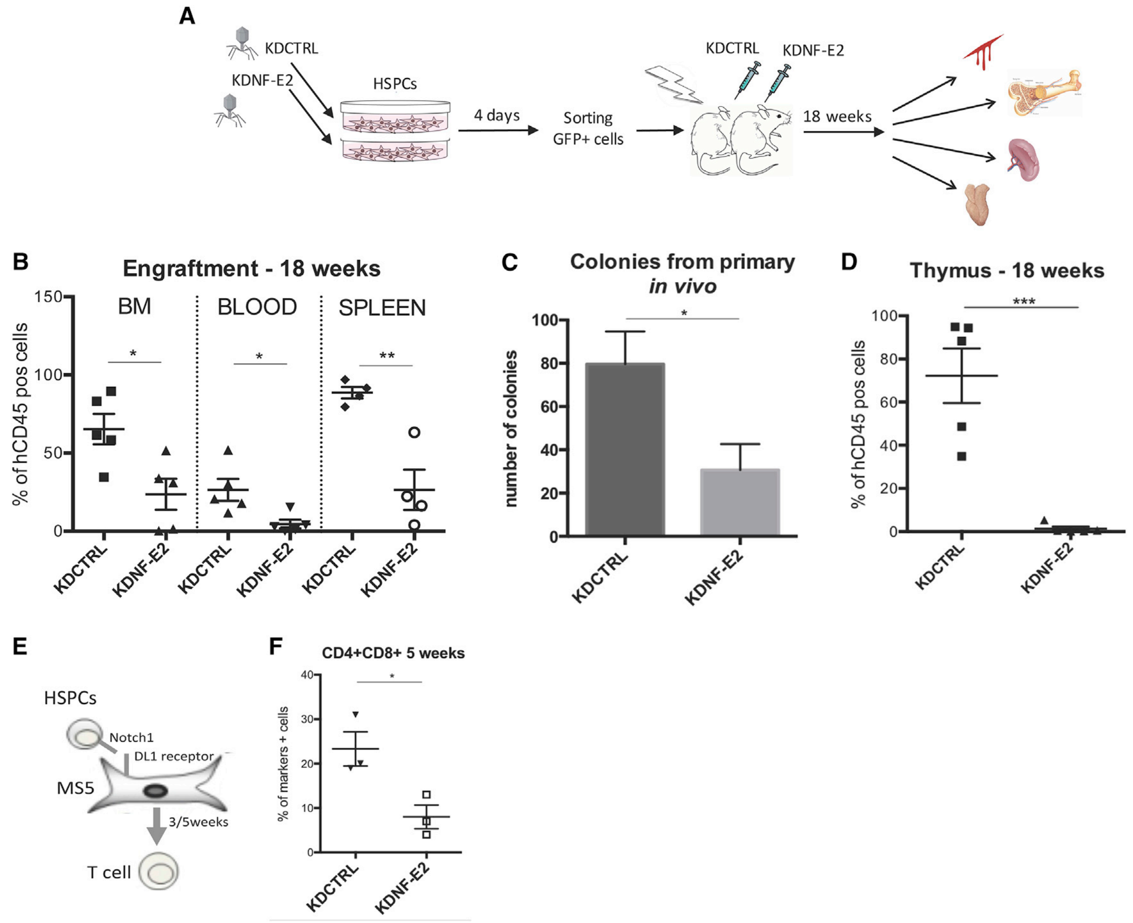


**Figure 2. Silencing *NF-E2* in HSPCs Affects Human HSC Self-Renewal and T Cell Differentiation *In Vivo***

(A) Scheme of HSPC transduction, injection into mice, and tissue analysis.

(B) Percentage of human cells in the BM (left), blood (middle), and spleen (right) of mice transplanted with transduced HSPCs. Each dot represents an individual mouse (KDCTRL, n = 5; KDNF-E2, n = 5).

(C) CFC assay using hCD45 cells taken from primary mice in (B).

(D) Percentage of human cells in the thymus of mice transplanted with transduced HSPCs. Each dot represents an individual mouse (KDCTRL, n = 5; KDNF-E2, n = 5).

(E) Scheme of T cell differentiation from HSPCs using MS5-DL1 feeder layer.

(F) Percentage of CD4/CD8 positive cells after 5 weeks of HSPC differentiation (n =3).

Error bars indicate the SEM of data from replicate experiments. The significance of the difference between samples was confirmed using unpaired t tests with equal SD. *$p < 0.05$, **$p < 0.001$, ***$p < 0.0001$. See also Figure S2.

from primary mice, and detected a significant decrease in the total number of CFUs (Figure 2C). Despite the still disputable role of NOTCH in mouse HSCs (Maillard et al., 2008; Varnum-Finney et al., 2011), the essential role of NOTCH1 in T cell differentiation is well recognized (Artavanis-Tsakonas et al., 1999). Thus, to demonstrate that the self-renewal impairment was based on defects in NOTCH1 activation, we studied the effect of NF-E2 in HSPC-derived T cells. Surprisingly, we detected an almost complete loss of human T cell engraftment in the thymus 18 weeks after silenced NF-E2 HSPC injection in mice (Figures 2D and S2B). To confirm these results, we used an established *in vitro* system for the assessment of T cell development (Calvo et al., 2012) (Figure 2E), through which we observed a strong reduction of pre-T cells at week 3 (Figure S2C, left panel) and double-positive CD4/CD8 cells at week 5 (Figures 2F and S2C, right panel).

In summary, we reveal that NF-E2 affects human hematopoiesis at two decision points, favoring differentiation over self-renewal and impairing the lymphoid lineage outcome. NF-E2 therefore offers an attractive target for stem cell manipulation strategies, particularly in the

context of immunodeficiency and acquired immunodeficiency syndrome.

## EXPERIMENTAL PROCEDURES

### Cell Lines

MOLT4 cell line was obtained from the Francis Crick Institute Cell Bank and were cultured in RPMI-1640 containing 10% heat-inactivated fetal bovine serum (FBS), 2 mmol/L penicillin, and streptomycin at 37°C in 5% $CO_2$/95% air. MS5 cells were obtained from the Francis Crick Institute Cell Bank and were cultured Iscove's modified Dulbecco's medium containing 10% heat-inactivated FBS, 2 mmol/L penicillin, and streptomycin at 37°C in 5% $CO_2$/95% air. They were subcultured upon reaching 80% confluence and selected using a mouse Sca-1 antibody (BD Pharmingen). γ-Secretase inhibitor DAPT (565770, CalbioChem) and compound XX (565789, CalbioChem) were added at a concentration of 10 μM and kept in culture for 3 days. MS5-DL1 cells were generated via lentiviral infection with pTRIP-EF1a-DL1-GFP vector, a kind gift of Dr. Pflumio. After infection, GFP-positive cells were flow sorted and plated for co-culture experiments.

### Lentiviral Production and Transduction

Lentivirus was produced from these constructs by transient calcium phosphate-mediated transfection of 293T cells with the lentiviral backbone plasmid, a VSV-g encoding plasmid, and a gag/pol plasmid. Virus supernatants were concentrated by ultracentrifugation to achieve titers of $1 \times 10^9$ IU/mL HSPCs ($CD34^+$ $CD38^-$ cells) were sorted from UCB $hCD34^+$ cells. Cells ($10^6$/mL) were pre-stimulated for 4–6 hr in serum-free medium (StemSpan, STEMCELL Technologies) with 150 ng/mL stem cell factor(SCF) (PeproTech), 150 ng/mL Flt3 ligand (PeproTech), 10 ng/mL interleukin-6 (IL-6) (PeproTech), 25 ng/mL granulocyte colony-stimulating factor (GCSF) (PeproTech), 20 ng/mL thrombopoietin (TPO) (PeproTech), 1% HEPES (Sigma-Aldrich), and 2 mmol/L penicillin and streptomycin (pen/strep; Sigma-Aldrich). Lentiviral infection was performed overnight using an MOI of 30. After 24 hr, cells were washed and seeded at $0.5 \times 10^5$ cells/mL in expansion medium (StemSpan with 300 ng/mL SCF, 300 ng/mL Flt3, 20 ng/mL TPO, 1% HEPES, and 1% pen/strep). Three days later, viable $GFP^+$ cells were sorted and transplanted into NSG mice.

### Flow Cytometry and Cell Sorting

For analysis and sorting of HSPCs derived from hUCB, cells were stained with hCD45-PeCy7, mCD45-PerCP5.5, Lineage-APC (eBioscience), CD34-PerCP, and CD38-APC780. Analysis of different lineage human engrafted cells in mice was assessed using CD19-APC, CD33-APC, CD33-PE, CD3-PEhCD45-PeCy7, and mCD45-PerCP5.5. Analysis of human T cells from engrafted mice was assessed using hCD45-PeCy7, mCD45-PerCP5.5, CD3-APC, CD4-PE, and CD8-PeCy7 and from *in vitro* culture using hCD45-PeCy7, CD34-APC, CD7-PE (or CD8-PE), and CD4-PerCP5.5. Finally, assessment of megakaryocyte numbers was done using CD41a-APC and CD42a-PerCP. Non-viable cells were excluded by DAPI staining. Appropriate isotype-matched antibodies were used as controls. All antibodies, unless specified, were purchased from BD Bioscience. Flow-cytometry analysis was performed using an LSRII flow cytometer. Cell sorting was performed using a FACSAria or INFLUX.

### ImageStreamX Sample Preparation and Analysis

MOLT4 cells were stained with hCD45-PeCy7. They were washed with PBS and fixed at 37°C for 15 min with 4% paraformaldehyde. They were then washed and resuspended in 2% Triton X-100 (Sigma-Aldrich) for 15 min and stained with NICD (ab8925, Abcam) for 60 min before selected secondary antibody was added for another 60 min. DAPI was added before analysis. Samples were acquired on an ASSIST calibrated Dual Camera, 12-Channel ImageStreamX Mark II Imaging Flow Cytometer (Amnis, Merck Millipore) using INSPIRE software (Amnis, Merck Millipore). Images were collected using a 60× objective lens and samples were acquired on a low-speed and high-sensitivity setting. Fluorescence was measured from a 405, 488, 561, and 642 nm laser for which laser powers were set to 20, 20, 80, and 50 mW, respectively. A dot-plot of Area versus Aspect ratio for Brightfield (Ch01) was gated to exclude debris during data acquisition.

Single-stained cells were acquired and a compensation matrix created, and the acquired data were compensated and analyzed using IDEAS software (Amnis, Merck Millipore). A threshold was set to include in the analysis all the acquired single nucleated cells. In particular, gates were set to include single, focused cells and then gated accordingly based on fluorescence. The mask, which defines the region of interest based on pixels within the cell image, was adapted from the default mask (M07) for the Nucleus (DAPI). The mask was adapted as follows: Threshold (M07, Ch07 DAPI, 50%). The Similarity feature was used to assess the co-localization of NICD APC (Ch11) and the Nucleus DAPI (Ch07) on selected cell populations; the following feature with the adapted mask was used to measure where co-localization occurs: Similarity Threshold (M07, Ch07 DAPI, 50%)_Ch07 DAPI_Ch11 APC.

### Megakaryocyte and T Cell Differentiation Media

Transduced HSPCs were sorted at day 4 as previously described and then seeded in megakaryocyte or T cell differentiation medium. Megakaryocyte differentiation: 500 cells/100 μL StemSpan with 50 ng/mL SCF, 50 ng/mL Flt3, 50 ng/mL IL-3, 100 ng/mL TPO, 1% HEPES, and 2 mmol/L pen/strep. Half-medium change was performed 1 week later and final analysis was performed after 2 weeks of T cell differentiation: 500 cells/100 μL were plated on pre-irradiated DL1-MS5 in a-MEM (Gibco) with 50 ng/mL SCF, 20 ng/mL Flt3, 10 ng/mL IL-7, and 200 nM insulin (Sigma-Aldrich). Half-medium change was performed every week for 4 weeks and analysis was performed at weeks 3 and 5.

### Colony Assay

Transduced HSPCs were sorted at day 4 as previously described and then seeded in methylcellulose medium (250 cells/mL) (H4434, STEMCELL) and incubated for 14 days at 37°C with 5% $CO_2$. Colonies were counted blinded to the experimental conditions. For secondary colonies 10,000 cells/mL were seeded after collection from primary colonies. For colonies from primary mice, 20,000 cells/well were seeded. All assays were performed in triplicates.

## Cell Cycle

Intracellular immunostaining for Ki67 was used to determine the cell-cycle status. Cells were washed with PBS, fixed in 1 mL of PBS with 2% methanol-free formaldehyde at room temperature for 10 min, and washed twice with PBS. They were then permeabilized with 1 mL of PBS containing 0.1%Triton X-100 (TX; Sigma) for 10 min at room temperature. After washing, cells were incubated with Alexa Fluor 647-Ki67 antibody (eBioscience) at 4°C for 1 hr. Cells were then resuspended with PBS 2% FBS buffer containing DAPI (2 mg/mL) and analyzed by FACS.

## mRNA Quantification

RNA was extracted using the RNeasy Micro Kit (Qiagen) and treated with DNase (Sigma). Reverse transcription was performed using the SensiScript Kit (Qiagen) according to the manufacturer's instructions. For real-time qPCR, SYBR Green master mix reagent (Applied Biosystems) was used and amplification was quantified using the ABI Prism 7700 sequence detection system (Applied Biosystems). To avoid possible amplification of contaminating DNA and unprocessed mRNA, we designed primers to anneal at the end of two exons separated by an intron.

Primers are as follows.

NF-E2: forward (FW) 5′-cct gct gtg act cca cca ca-3′, reverse (REV) 5′-gcc aga gtc tgg tcc agg t-3′
HES1: FW 5′-tgg aaa tga cag tga agc acc t-3′, REV 5′-gtt cat gca ctc gct gaa gc-3′
P21: FW 5′-cct gtc act gtc ttc tac cct t-3′, REV 5′-aga aga tca gcc ggc gtt t-3′
PSEN2: FW 5′-ccg ctg cta caa ggt tca tcc-3′, REV 5′-tcc aga cag tca gca aga gg-3′
NCSTN: FW 5′-ctg tgt tcg cct gct caa c-3′, REV 5′-ggg cca tca gtc aat acc ca-3′
APH1A: FW 5′-acc tac tga cat cgg gac tg-3′, REV 5′-gag gct gcg ctg aat act tc-3′
PSNEN: FW 5′-acc tgt gcc gga agt act ac-3′, REV 5′-ctg ttc tgt gta ggc tgg ga-3′
b-ACT: FW 5′-aca gag cct cgc ctt tgc-3′, REV 5′-cac gat gga ggg gaa gac-3′

## Tissue Extraction

BM cells were extracted by crushing leg bones in a mortar. Spleen and thymus were strained through a 70-μm Falcon Cell Strainer (Fisher Scientific). Cells were resuspended in PBS 2% FBS.

## Western Blot Analysis

Total protein extracts (20 μg) were run on a denaturing 10% SDS-PAGE gel and transferred by wet transfer to nitrocellulose membranes. The primary antibodies used were NF-E2 (sc-291, Santa Cruz Biotechnology), b-ACT (A5441, Sigma-Aldrich), NICD for western (2421, Cell Signaling, Figure 1H and ab8925, Abcam Figure S1A) and for image streaming (ab8925, Abcam), and p21 (sc-397, Santa Cruz). Protein bands were visualized using an enhanced chemiluminescence visualization system (ECL Plus, Amersham, Life Sciences).

## hCD34$^+$ Cell Isolation

UCB samples were obtained from normal full-term deliveries after signed informed consent. UCB sample collection was approved by the East London Ethical Committee and in accordance with the Declaration of Helsinki. Mononuclear cells (MNCs) were purified by Ficoll-Paque density centrifugation (GE Healthcare Life Sciences) followed by ammonium chloride red cell lysis. Density-separated cord blood mononuclear cells were magnetically sorted for CD34 positivity via the StemSep system (STEMCELL) according to the manufacturer's instructions.

## Transplantation in NSG Mice

Eight- to 12-week-old NOD-SCID IL2Rγnull (NSG) mice (originally obtained from The Jackson Laboratory) were used. Twenty-four hours before transplantation, mice were sublethally irradiated at the dose of 3.75 Gy. Cells were injected using intravenous injection ($1 \times 10^4$ cells/mouse). To assess the level of engraftment, BM samples were aspirated from a single long bone at different time points after transplantation (8, 12, and 16 weeks). All animal work was done in accordance with the UK Home Office and CRUK guidelines.

## RNA Silencing

For silencing of *NF-E2* expression, a lentiviral vector (CS-RfA-EG) was engineered to express two 19-bp nucleotide short hairpin RNAs (shRNAs) against NF-E2 (5′-TGA GGG ATA ATC CTG AGT C-3′ and 5′-GAA CTG ACT TGG CAG GAG A-3′) and one shRNA against luciferase used as a control (5′-ACG CTG AGT ACT TCG AAA T-3′).

## Statistical Analysis

Statistical analyses were performed using GraphPad Prism Version 6.0f (GraphPad Software). Error bars indicate the SEM of data from replicate experiments. The significance of difference between samples within figures was confirmed using unpaired t tests with equal SD. Observed differences were regarded as statistically significant if the calculated two-sided p value was less than 0.05.

## SUPPLEMENTAL INFORMATION

Supplemental Information includes two figures and can be found with this article online at http://dx.doi.org/10.1016/j.stemcr.2017.05.027.

## AUTHOR CONTRIBUTIONS

A.d.T. was involved with all aspects of the study's design, execution, analysis, and manuscript preparation. D.P., K.R.-P., and S.P. contributed to experiments and analysis. D.B. contributed to design, analysis, and manuscript preparation as well as providing project leadership. All authors reviewed the manuscript during its preparation.


## ACKNOWLEDGMENTS

We wish to thank Françoise Pflumio for sharing the pTRIP-EF1a-DL1-GFP plasmid. We also gratefully acknowledge William Gray for critical reading of the manuscript. We thank the Biological Resource Unit (BRU) and the Flow Cytometry, *In Vivo* Imaging, and Light Microscopy core facilities at the Francis Crick Institute. We would like to thank Mrs. Shohreh Beski (Obstetric Department, Royal London Hospital, London, UK) and her team as well as

Mrs. Jashu Patel for helping in the collection of UCB. This work was supported by the Francis Crick Institute, which receives its core funding from Cancer Research UK (FC0010045), the UK Medical Research Council (FC0010045), and the Wellcome Trust (FC001045).

Received: March 31, 2017
Revised: May 22, 2017
Accepted: May 23, 2017
Published: June 22, 2017

## REFERENCES

Andrews, N.C., Erdjument-Bromage, H., Davidson, M.B., Tempst, P., and Orkin, S.H. (1993). Erythroid transcription factor NF-E2 is a haematopoietic-specific basic-leucine zipper protein. Nature *362*, 722–728.

Artavanis-Tsakonas, S., Rand, M.D., and Lake, R.J. (1999). Notch signaling: cell fate control and signal integration in development. Science *284*, 770–776.

Bagger, F.O., Rapin, N., Theilgaard-Monch, K., Kaczkowski, B., Thoren, L.A., Jendholm, J., Winther, O., and Porse, B.T. (2013). HemaExplorer: a database of mRNA expression profiles in normal and malignant haematopoiesis. Nucleic Acids Res. *41*, D1034–D1039.

Calvo, J., BenYoucef, A., Baijer, J., Rouyez, M.C., and Pflumio, F. (2012). Assessment of human multi-potent hematopoietic stem/progenitor cell potential using a single in vitro screening system. PLoS One *7*, e50495.

Di Tullio, A., Vu Manh, T.P., Schubert, A., Castellano, G., Mansson, R., and Graf, T. (2011). CCAAT/enhancer binding protein alpha (C/EBP(alpha))-induced transdifferentiation of pre-B cells into macrophages involves no overt retrodifferentiation. Proc. Natl. Acad. Sci. USA *108*, 17016–17021.

Goerttler, P.S., Steimle, C., Marz, E., Johansson, P.L., Andreasson, B., Griesshammer, M., Gisslinger, H., Heimpel, H., and Pahl, H.L. (2005). The Jak2V617F mutation, PRV-1 overexpression, and EEC formation define a similar cohort of MPD patients. Blood *106*, 2862–2864.

Kaufmann, K.B., Grunder, A., Hadlich, T., Wehrle, J., Gothwal, M., Bogeska, R., Seeger, T.S., Kayser, S., Pham, K.B., Jutzi, J.S., et al. (2012). A novel murine model of myeloproliferative disorders generated by overexpression of the transcription factor NF-E2. J. Exp. Med. *209*, 35–50.

Lecine, P., Blank, V., and Shivdasani, R. (1998). Characterization of the hematopoietic transcription factor NF-E2 in primary murine megakaryocytes. J. Biol. Chem. *273*, 7572–7578.

Maillard, I., Koch, U., Dumortier, A., Shestova, O., Xu, L., Sai, H., Pross, S.E., Aster, J.C., Bhandoola, A., Radtke, F., and Pear, W.S. (2008). Canonical notch signaling is dispensable for the maintenance of adult hematopoietic stem cells. Cell Stem Cell *2*, 356–366.

Motohashi, H., Kimura, M., Fujita, R., Inoue, A., Pan, X., Takayama, M., Katsuoka, F., Aburatani, H., Bresnick, E.H., and Yamamoto, M. (2010). NF-E2 domination over Nrf2 promotes ROS accumulation and megakaryocytic maturation. Blood *115*, 677–686.

Mutschler, M., Magin, A.S., Buerge, M., Roelz, R., Schanne, D.H., Will, B., Pilz, I.H., Migliaccio, A.R., and Pahl, H.L. (2009). NF-E2 overexpression delays erythroid maturation and increases erythrocyte production. Br. J. Haematol. *146*, 203–217.

Shivdasani, R.A., Rosenblatt, M.F., Zucker-Franklin, D., Jackson, C.W., Hunt, P., Saris, C.J., and Orkin, S.H. (1995). Transcription factor NF-E2 is required for platelet formation independent of the actions of thrombopoietin/MGDF in megakaryocyte development. Cell *81*, 695–704.

Stier, S., Cheng, T., Dombkowski, D., Carlesso, N., and Scadden, D.T. (2002). Notch1 activation increases hematopoietic stem cell self-renewal in vivo and favors lymphoid over myeloid lineage outcome. Blood *99*, 2369–2378.

Tsai, J.J., Dudakov, J.A., Takahashi, K., Shieh, J.H., Velardi, E., Holland, A.M., Singer, N.V., West, M.L., Smith, O.M., Young, L.F., et al. (2013). Nrf2 regulates haematopoietic stem cell function. Nat. Cell Biol. *15*, 309–316.

Varnum-Finney, B., Halasz, L.M., Sun, M., Gridley, T., Radtke, F., and Bernstein, I.D. (2011). Notch2 governs the rate of generation of mouse long- and short-term repopulating stem cells. J. Clin. Invest. *121*, 1207–1216.

Wang, W., Schwemmers, S., Hexner, E.O., and Pahl, H.L. (2010). AML1 is overexpressed in patients with myeloproliferative neoplasms and mediates JAK2V617F-independent overexpression of NF-E2. Blood *116*, 254–266.

Stem Cell Reports, Volume *9*

## Supplemental Information

# Nuclear Factor Erythroid 2 Regulates Human HSC Self-Renewal and T Cell Differentiation by Preventing NOTCH1 Activation

**Alessandro Di Tullio, Diana Passaro, Kevin Rouault-Pierre, Sukhveer Purewal, and Dominique Bonnet**

# Figure S1

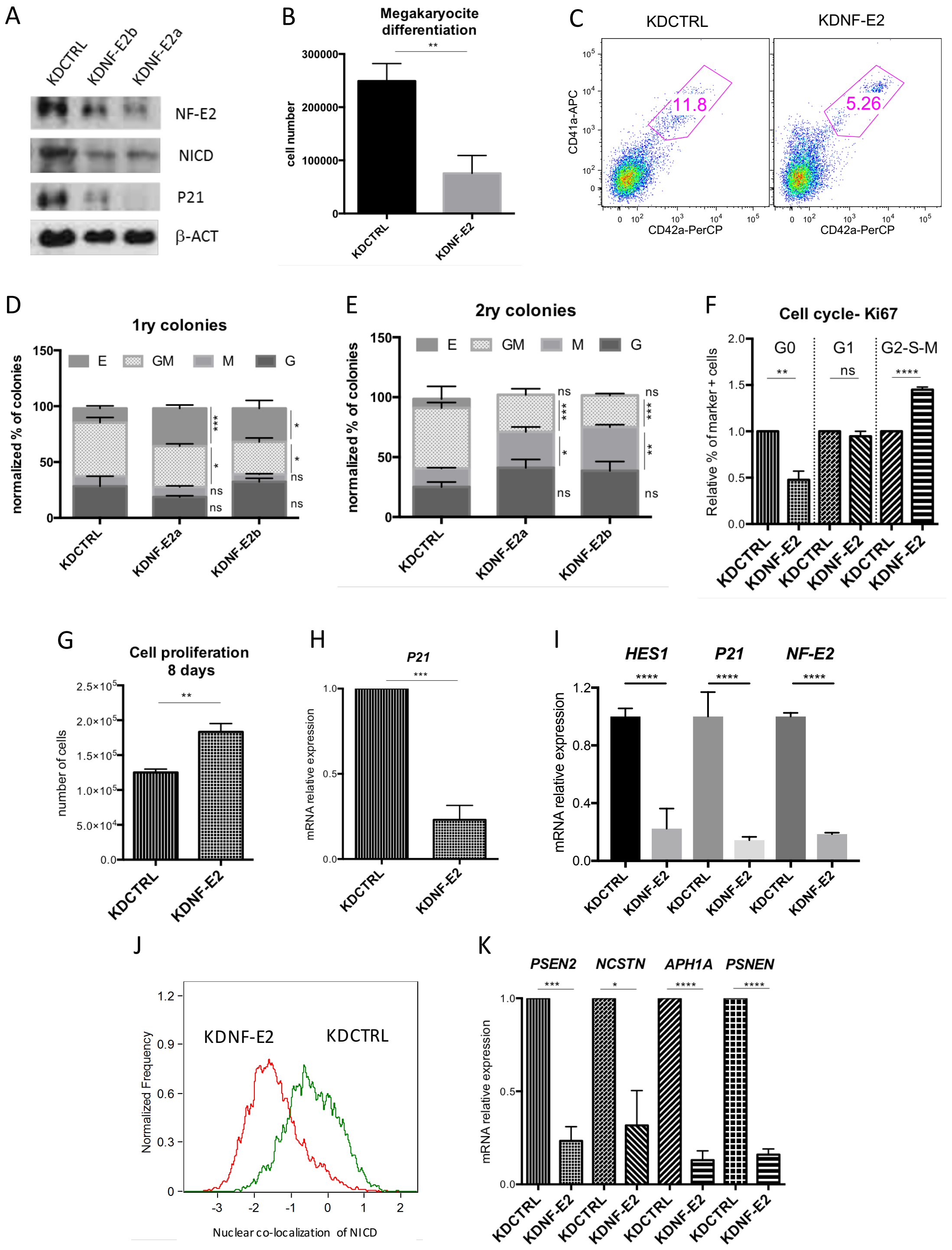
A
KDCTRL
KDNF-E2b
KDNF-E2a
NF-E2
NICD
P21
β-ACT
B
Megakaryocite differentiation
cell number
KDCTRL
KDNF-E2
C
KDCTRL
KDNF-E2
CD41a-APC
CD42a-PerCP
11.8
5.26
D
1ry colonies
normalized % of colonies
E
GM
M
G
KDCTRL
KDNF-E2a
KDNF-E2b
E
2ry colonies
normalized % of colonies
F
Cell cycle- Ki67
Relative % of marker + cells
G0
G1
G2-S-M
G
Cell proliferation 8 days
number of cells
H
P21
mRNA relative expression
I
HES1
P21
NF-E2
mRNA relative expression
J
KDNF-E2
KDCTRL
Normalized Frequency
Nuclear co-localization of NICD
K
PSEN2
NCSTN
APH1A
PSNEN
mRNA relative expression

**Figure S1. Knock-down of *NF-E2* in HSPCs reduces megakaryocyte differentiation and favors cell proliferation over self-renewal by inhibition of NOTCH1 activation, Related to Figure 1.**

**(A)** Western Blot showing the expression of P21, Notch 1 NCID and NF-E2 in HSPCs 6 days after transduction. *β-ACT* has been used as a control.

**(B)** Quantification of $CD41a^+CD42a^+$ cells in transduced HSPCs 2 weeks after the beginning of megakaryocyte differentiation. **(C)** FACS analysis showing the percentage of CD41a and CD42a as in A. **(D)** CFC assay using HSPCs transduced with KDCRTL or two independent KDNF-E2 (N=3. **(E)** Secondary CFC assay using cells taken from CFU in C (N=3). **(F)** Cell cycle analysis by FACS using Ki67 and DAPI to identify proportion of cells in G0, G1 and S/G2/M phases of HSPCs 8 days after transduction (N=3). **(G)** Quantification of cell proliferation by cell counting of HSPCs 8 days after transduction (N=3). **(H)** RNA expression of *P21* in HSPCs 4 days after transduction. *β-ACT* was used as a control gene (N=3). **(I)** RNA expression of *HES1*, *P21*, and *NF-E2* in MOLT4 transduced with KDCTRL and KDNF-E2. *β-ACT* was used as a control gene (N=3). **(J)** $ImageStream^x$ analysis showing the frequency of NICD nuclear co-localization in KDNF-E2 (red) and KDCTRL (green) transduced MOLT4 cells as shown in Figure 1J. **(K)** RNA expression of *PSEN2*, *NCSTN*, *APH1A* and *PSNEN* in HSPCs 4 days after transduction. *β-ACT* was used as a control gene (N=3). ns= not significant, * $p<0.05$; ** $p<0.001$ and *** $p<0.0001$.

## Figure S2

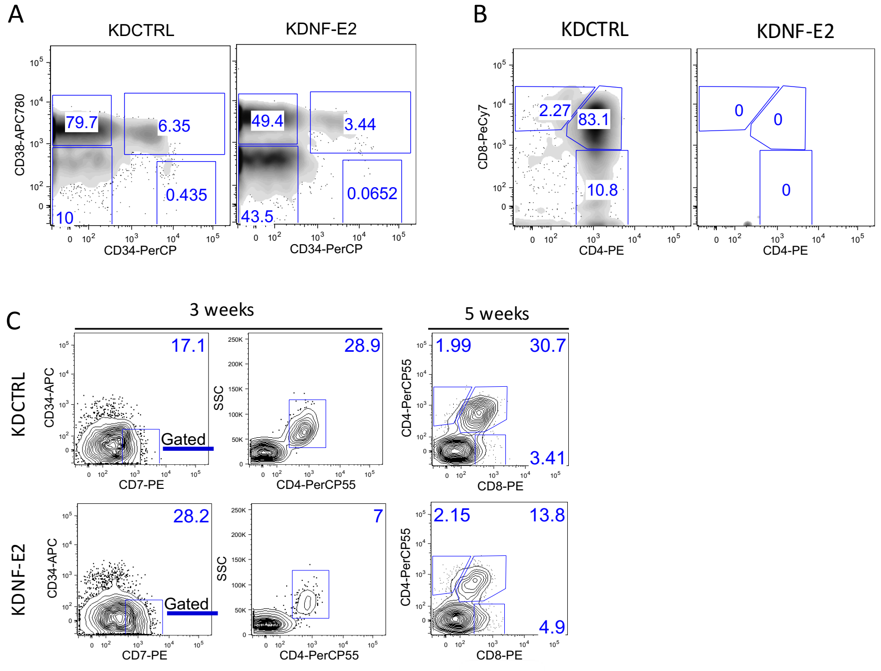


**Figure S2. Knock-down of *NF-E2* in HSPCs impairs T cell development, Related to Figure 2.**

**(A)** FACS analysis showing the percentage of human CD38/CD34 cells in the bone marrow of mice transplanted with transduced HSPCs at 18 weeks after injection (N=5). **(B)** Representative FACS analysis showing the percentage of human CD4 and CD8 cells in thymus of mice transplanted with transduced HSPCs at 18 weeks after injection. **(C)** Representative FACS analysis showing the percentage of pre-T cell makers 3 weeks (left panel) and mature T cell markers 5 weeks (right panel) after the induction of T cell differentiation.
